# Supplementary material for: Transcriptome Analysis Reveals Vimentin-Induced Disruption of Cell–Cell Associations Augments Breast Cancer Cell Migration
Source: Cells. 2022 Dec 13;11(24):4035. doi: 10.3390/cells11244035 (PMC9776984; doi:10.3390/cells11244035)
Supplement: Supplementary file 1 [file cells-11-04035-s001.zip › DEGs and enrichment analysis.zip!/DEGs and enrichment analysis/WT_VIMvsVector GO enrichment/UP/WT_VIMvsVector_up.MFbar.pdf]

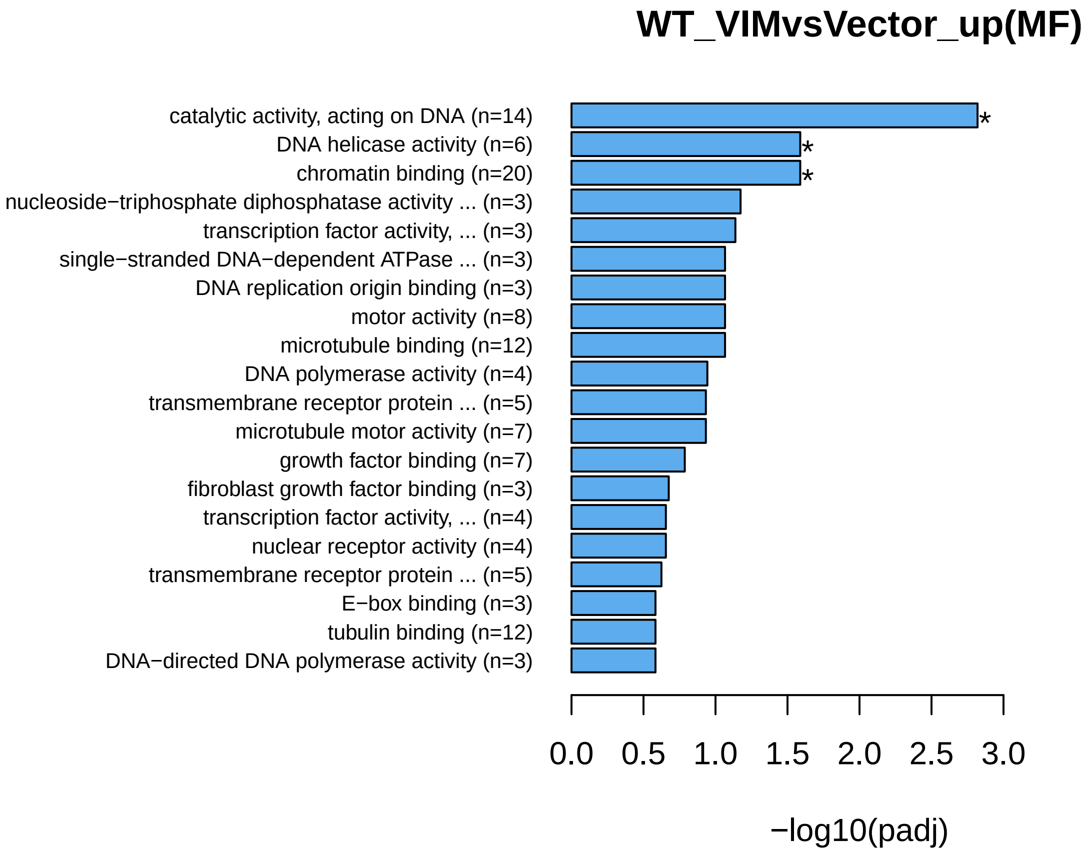
WT_VIMvsVector_up(MF)
catalytic activity, acting on DNA (n=14)
DNA helicase activity (n=6)
chromatin binding (n=20)
nucleoside−triphosphate diphosphatase activity ... (n=3)
transcription factor activity, ... (n=3)
single−stranded DNA−dependent ATPase ... (n=3)
DNA replication origin binding (n=3)
motor activity (n=8)
microtubule binding (n=12)
DNA polymerase activity (n=4)
transmembrane receptor protein ... (n=5)
microtubule motor activity (n=7)
growth factor binding (n=7)
fibroblast growth factor binding (n=3)
transcription factor activity, ... (n=4)
nuclear receptor activity (n=4)
transmembrane receptor protein ... (n=5)
E−box binding (n=3)
tubulin binding (n=12)
DNA−directed DNA polymerase activity (n=3)
*
*
*
0.0
0.5
1.0
1.5
2.0
2.5
3.0
−log10(padj)
